# Cervicogenic dizziness alleviation after coblation discoplasty: a retrospective study

**DOI:** 10.1080/07853890.2021.1910336

**Published:** 2021-04-15

**Authors:** Liang-liang He, Ru-jing Lai, Jacqueline Leff, Rong Yuan, Jian-ning Yue, Jia-xiang Ni, Li-qiang Yang

**Affiliations:** a Department of Pain Management, Xuanwu Hospital, Capital Medical University, Beijing, China; b Department of Anesthesiology, Longyan First Hospital, Affiliated to Fujian Medical University, Longyan City, Fujian, China; c Touro University College of Medicine, New York, NY, USA; d Ultrasonic Diagnosis Department, North District of Peking University Third Hospital, Beijing, China

**Keywords:** Cervicogenic dizziness, cervicogenic vertigo, coblation discoplasty, coblation nucleoplasty

## Abstract

**Objective:**

Little is known about the therapeutic relationship between coblation discoplasty and cervicogenic dizziness (CGD). CGD can be caused by abnormal proprioceptive inputs from compressed nerve roots, intradiscal mechanoreceptors and nociceptors to the vestibulospinal nucleus in the degenerative cervical disc. The aim was to analyze the efficacy of coblation discoplasty in CGD through intradiscal nerve ablation and disc decompression in a 12-month follow-up retrospective study.

**Methods:**

From 2015 to 2019, 42 CGD patients who received coblation discolplasty were recruited as the surgery group, and 22 CGD patients who rejected surgery were recruited as the conservative group. Using intent-to-treat (ITT) analysis, we retrospectively analyzed the CGD visual analogue scale (VAS), neck pain VAS, CGD frequency score, and the CGD alleviation rating throughout a 12-month follow-up period.

**Results:**

Compared with conservative intervention, coblation discoplasty revealed a better recovery trend with effect sizes of 1.76, 2.15, 0.92, 0.78 and 0.81 in CGD VAS, and effect sizes of 1.32, 1.54, 0.93, 0.86 and 0.76in neck pain VAS at post-operative 1 week, and 1, 3, 6, 12 months, respectively. The lower CGD frequency score indicated fewer attacks of dizziness until postoperative 3 months (*p* < 0.01). At post-operative 12 months, the coblation procedure showed increased satisfactory outcomes of CGD alleviation rating (*p* < .001, −1.00 of effect size).

**Conclusions:**

Coblation discoplasty significantly improves the severity and frequency of CGD, which is important inbridging unresponsive conservative intervention and open surgery.

**Key messages**
- There is a correlation between the degenerative cervical disc and cervicogenic dizziness (CGD).
- CGD can be caused by abnormal proprioceptive inputs from a compressed nerve root and intradiscal mechanoreceptors and nociceptors to the vestibulospinal nucleus in the degenerative cervical disc.
- Cervical coblation discoplasty can alleviate CGD through ablating intradiscal nerve endings and decompressing the nerve root.

## Introduction

Cervicogenic dizziness (CGD) is characterized by the presence of “lightheadedness or disequilibrium” associated with cervical dysfunction [1,2]. This is not to be confused with the “rotatory” sensation arising from vestibular pathology [3]. The aetiology of CGD is attributed to abnormal proprioceptive inputs from cervical proprioceptors into the ipsilateral medial and inferior vestibulospinal nucleus (cervico-vestibular pathway), which in turn disrupts the head and neck’s orientation in space [4–6]. Studies report that the incidence of dizziness in patients with cervical spondylosis is ∼50% [7], and up to 65% in patients aged over 65 years [8]. Surprisingly, up to 89% of dizziness in 1000 outpatients [1], was diagnosed as CGD, which negatively affects quality of life.

**Figure 1.**
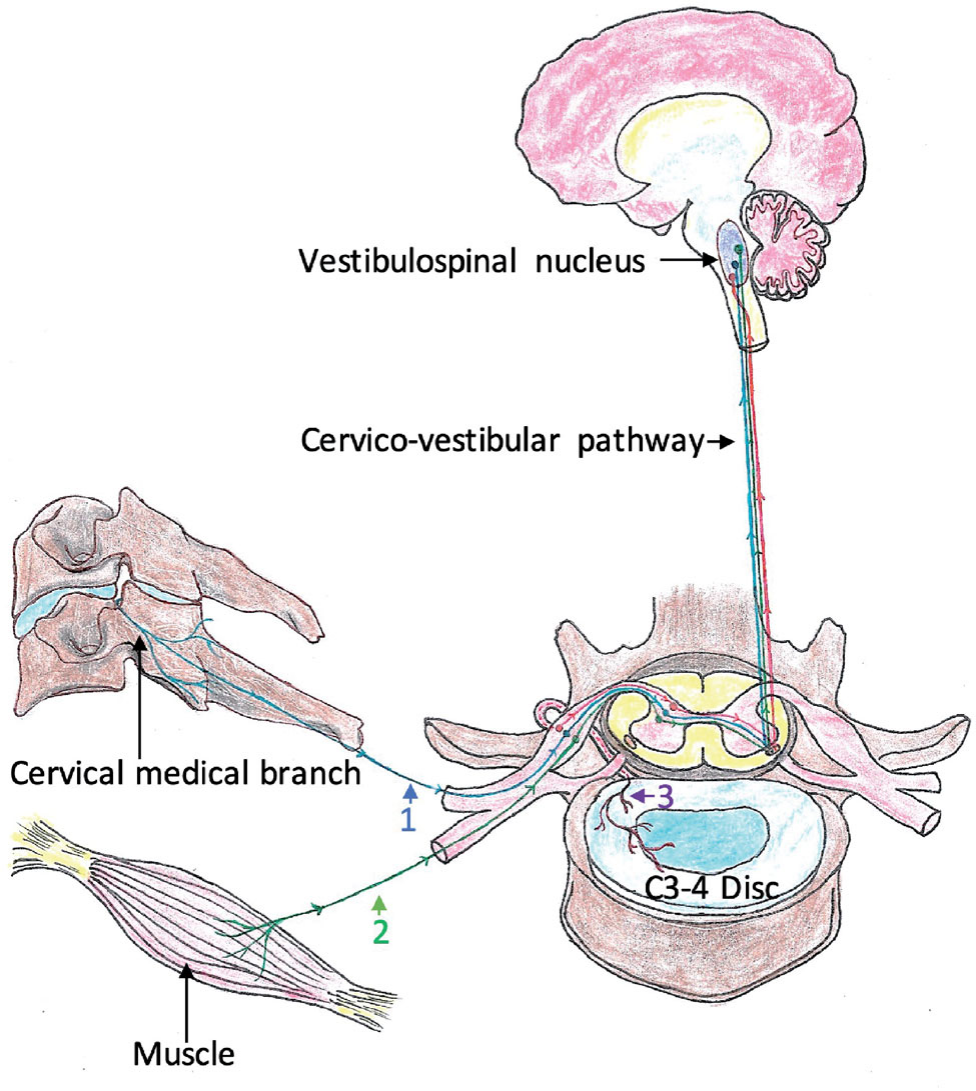
The pathological pathway of cervical dizziness between cervical structures and vestibulospinal nucleus. Pathway 1: originating from mechanoreceptors in the facet joint; pathway 2: originating from muscle spindles; pathway 3: originating from mechanoreceptors in intradiscal endings.

Abnormal afferent inputs from the Ruffini corpuscles of the facet joints and the muscle spindles to the vestibulospinal nucleus in the degenerative cervical spine are considered as major pathological sources of CGD [9–11]. As the essential mechanoreceptive signal transport pathway in the cervico-vestibulo-cervical loop, cervical nerve root also possibly provokes CGD because of herniated cervical disc compression [5,12]. Besides, studies indicated that degenerative cervical discs have an abundant distribution of Ruffini corpuscles [13,14], which project abnormal proprioceptive inputs to C2–C8 of the dorsal root ganglion [15] and then reach the vestibulospinal nucleus [5]. (Figure 1) The contribution of degenerative cervical discs to CGD is further identified by desirable improvement following anterior cervical decompression and fusion [14,16,17].

**Figure 2.**
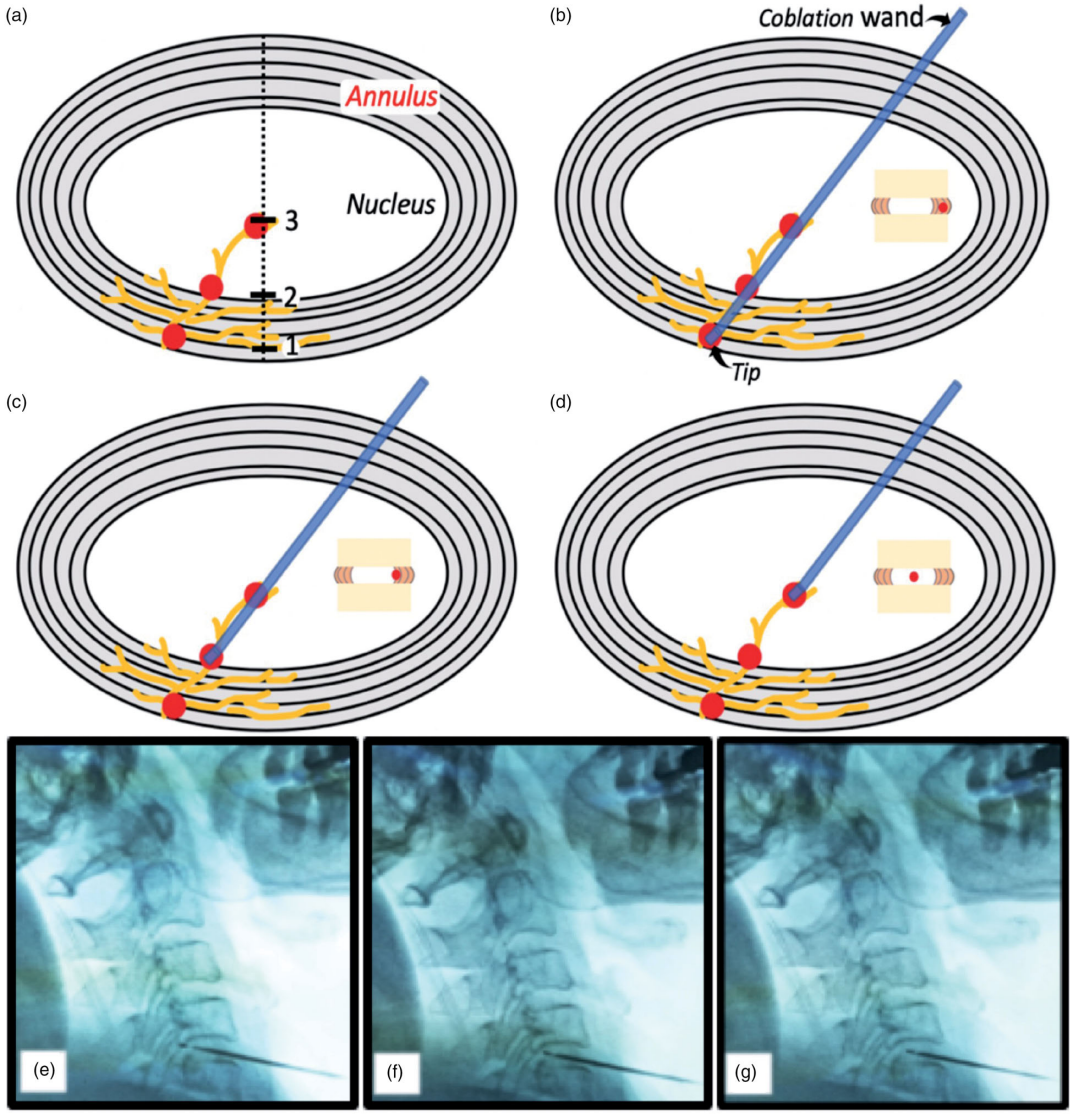
Three distinct ablation regions in the degenerative disc. (a) Mechanoreceptors innervate from annulus into nucleus, 1-plot indicates the margin of annulus, 2-plot indicates the boundary between annulus and nucleus, and 3-plot indicates the mid-nucleus; (b–d) Ablation region is in the margin of the annulus, the boundary between annulus and nucleus, and the mid-nucleus, respectively; (e–g) In C4–5 level, the ablation region is in the margin of the annulus, the boundary between annulus and nucleus, and the mid-nucleus under fluoroscopic guidance, respectively.

To bridge unresponsive conservative therapy and open surgery, minimally invasive intervention is recommended in accordance with stepwise therapy principles. In a retrospective study, Li et al. [18] found that cervical vertigo can benefit from coblation nucleoplasty. However, limitations included the lack of a control group and an intradiscal ablation region around the centre of nucleus, but not annulus which is innervated by Ruffini corpuscles at a high density [13,14]. Starting in 2015, the active portion of the ablation tip is deployed into both the annulus and nucleus (Figure 2(a)), which we named as coblaltion discoplasty, in our cervical coblation procedure set up [19]. Here we intended to retrospectively analyze 64 patients with CGD who underwent coblation discoplasty from May 2015 to January 2019, and to investigate whether CGD can be further alleviated compared with the conservative intervention approach.

## Materials and methods

### Characteristics and participants

This retrospective study was performed after obtaining approval from the Institution’s Ethics Examining Committee of Human Research. Between May 2015 and January 2019, 64 CGD patients with positive intradiscal injections of lidocaine were advised to receive cervical coblation discoplasty. Ages of patients ranged from 35 to 69 years old and duration/diagnosis of CGD from 1 to 11 years. Out of the 64 patients, the 42 patients that received coblation discoplasty were recruited as the surgery group (group S), while the remaining 22 patients who rejected surgery were recruited as the conservative group (group C).

Because “rotatory” sensation is closely linked to vestibular disease [20,21], CGD was accentuated by “lightheadedness or disequilibrium” sensations [1,2]. The inclusion criteria of coblation procedure was as follows: (1) met the diagnostic standard of CGD (Table 1); (2) the CGD severity of visual analogue scale (VAS) ≥4; (3) the CGD duration ≥ 6 months; (4) the neck pain VAS ≥4; (5) palpation pain or restrictive mobility of the cervical spine observed by physical examination; (6) cervical magnetic resonance imaging(MRI) showed contained herniation disc, not compromising more than 1/3 of the central spinal canal, the disc height ≥50% in comparison to normal adjacent discs; (7) one stepwise diagnostic procedure was performed to identify the source of CGD: trigger point injections to test for neck muscle spindles [9,22], cervical medial branch block to test for Ruffini corpuscles of the facet joint [10,11,23], cervical nerve root block to inhibit the transmission from peripheral abnormal inputs to the vestibular nucleus [5,12]. If short-term or no response to the above procedures were reported, intradiscal injection of 0.5 ml 0.25% of lidocaine to test positive disc (dizziness relief ≥50% after post injection, 1 day), because of the abundances of mechanoreceptor endings distribution inside [10,16].

**Table 1.** Inclusion criteria for cervicogenic dizziness.

Sensation: “lightheadedness or disequilibrium” not “rotatory”; Onset: gradual, but not acute; Episode: minutes to hours; Trigger: neck movement or specific neck position; Coexistence: neck pain and/or stiffness; Accompany symptom: headache, shoulder pain, and/or radicular pain occasionally; Physical examination: palpation for pain and tenderness, limited cervical range of motion; Intervention: manual therapy, trigger point injection, or nerve block of neck is positive

Patients with the following conditions were excluded: peripheral vestibular disorders(such as: benign paroxysmal position vertigo characterized by acute onset, lasting seconds to 1 min, positional, triggered by movement accompanied by nystagmus; Meniere’s disease characterized by general onset, lasting several hours, not positional, hearing loss, tinnitus, fullness; vestibular neuronitis characterized by acute onset, lasting several weeks, central vestibular disorders (such as: posterior circulation infraction characterized by sudden acute onset, ataxia, diplopia, cranial nerve defects, limb weakness; tumours characterized by general onset, brainstem and/or cerebellar dysfunction; vestibular migraine characterized by throbbing headache, vertigo, sensitivity to auditory or visual sensory stimulation, and oculomotor changes, bow hunter’s symptom, whiplash injury, progressive cervical myelopathy, spinal fracture, and coagulopathy.

In group S, the coblation discoplasty procedure was performed under fluoroscopic guidance with anterior-posterior and lateral views in an operating room under sterile conditions. Patients were placed in a supine position with slight hyperextension of the neck. A puncture was performed using the right or left anterior approach. The ablation wand tip was inserted into the disc and guided to the opposite posterior target lesion and annular outer margin, but not beyond the vertebral body posterior edge (Figure 2(b,e)). After confirming the tip was in a secure position without paresthaesia or abnormal movement following ½ sec of coagulation stimuli, ablation was conducted by rotating the wand 360° with level 2–3 of ablation power. Subsequently, during the withdrawal process along the puncture route, we continued to ablate two more regions: one boundary was between the annulus and nucleus (the midpoint of line linking the midpoint and posterior margin of disc lateral view) (Figure 2(c,f)), the other was the mid-nucleus (the midpoint of the disc lateral view) (Figure 2(d,g)). Following the procedures, all patients were advised to avoid long-term lowering of the head and to wear a cervical collar for 4 weeks. In group C, conservative treatment included physical therapy (TENS therapy), nonsteroidal anti-inflammatory drugs, muscle relaxants or nerve block injections.

In a 12-month follow-up, the primary outcome was to gauge the severity of CGD, which was measured with VAS from 0 to 10 (0 is rated as no severity and 10 worst severity). The secondary outcomes included CGD frequency score (0 = no dizziness; 1= dizziness less than once per month; 2 = 1–4 episodes per month; 3 = 1–4 episodes per week; 4 = dizziness once daily; 5 = dizziness more than once a day or constant); CGD alleviation rating (1–3: 1 is "complete remission"; 2 is "partial remission"; 3 is “no-remission”); and neck pain VAS from 0 to 10 (0 is rated as no severity and 10 worst severity).

### Statistical methods

All data was processed by GraphPad Prism software version 8.0, and statistical significance was declared at the level of *p* < .05 (2-tailed), effect size (Cohen’s d). Normally distributed continuous data on patient demographics and dizziness characteristics was reported as the mean ± standard deviation and calculated using independent samples t-tests, and categorical data were analyzed with the chi-squared test between the two groups. The intent-to-treat (ITT) analysis was conducted in the study, and the missing data with the last observation carried forward imputation method [24]. At different time points during follow-up, the comparison of the VAS and frequency between groups was analyzed with two-way repeated measures ANOVA. The CGD alleviation rating at post-operative 12th month was reported as the medium [25th quartile to 75th quartile] and calculated with the Mann–Whitney test.

## Results

### Participants

No differences in patients’ age, gender, or pain duration were found between the S and C groups in Table 2. In group S, because of unsatisfied therapeutic efficacy, a total number of 3 patients received anterior cervical decompression and fusion: one at post-operative month 3, one at month 6 and one at month 9. In group C, due to unbearable CGD, one patient received anterior cervical decompression and fusion at post-conservative treatment month 5. Five patients opted to receive coblation discoplasty: one at 1 month, two at 4 months, one at 7 months, and one at 10 months.

**Table 2.** Demographic data of patients in CGD.

	*N*	Age (y)	Gender, *n* (%)		Duration (y)	Diagnostic level, *n* (%)			
			Male	Female		C3/4	C4/5	C5/6	C6/7
Group-S	42	55 ± 9	18 (43)	24 (57)	4.4 ± 2.3	7 (16)	13 (31)	17 (40)	5 (12)
Group-C	22	53 ± 6	10 (45)	12 (54)	3.8 ± 1.3	4 (18)	8 (36)	8 (36)	2 (9)

### CGD VAS

**Figure 3.**
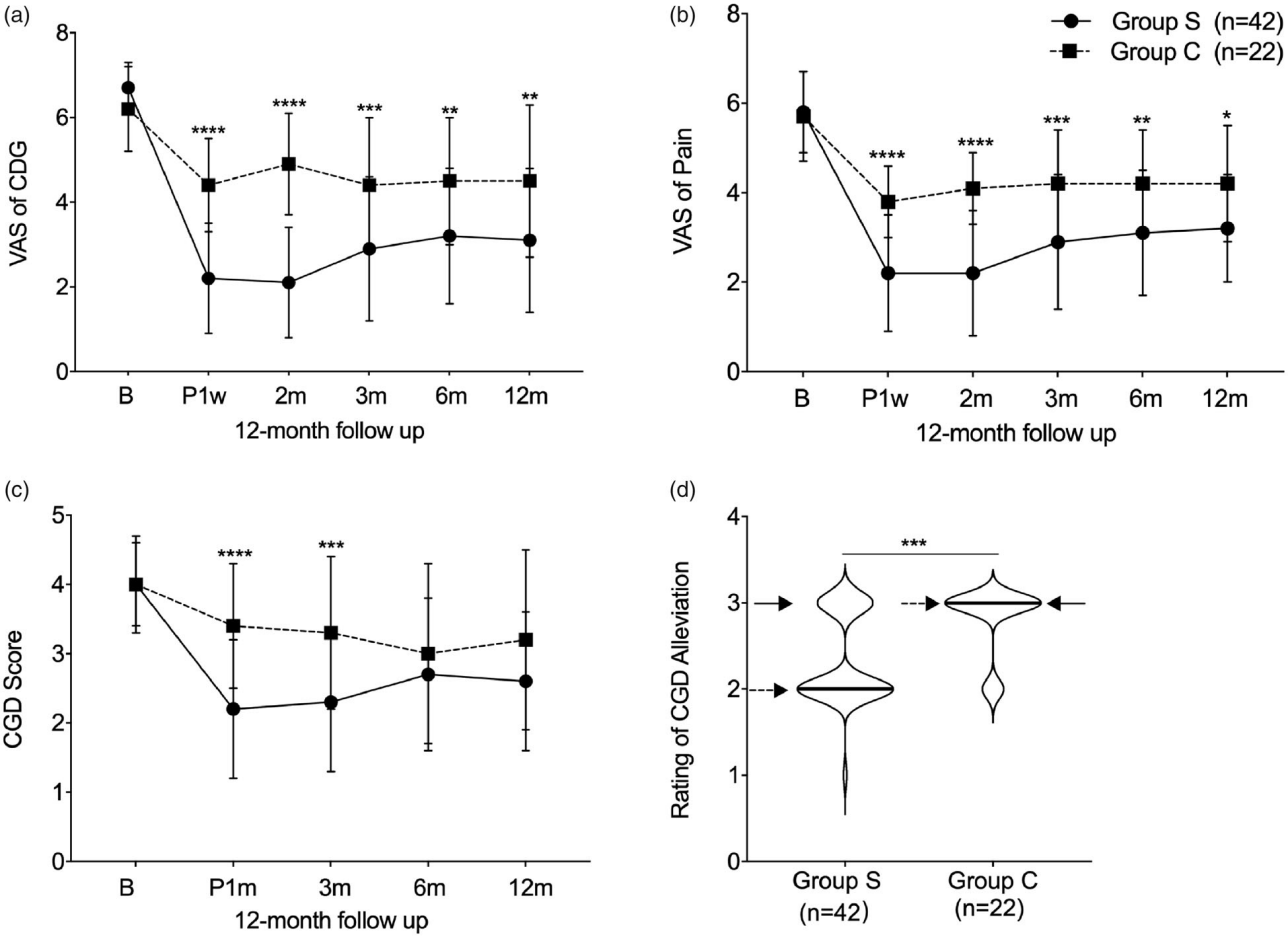
The efficacy outcomes after coblationdiscoplasty in 12 months follow-up. (a) Comparison of CDG VAS between two groups; (b) Comparison of neck pain VAS between two groups; (c) Comparison of CGD frequency score between two groups; (d) Comparison of CGD alleviation rating between two groups. B: baseline; P: post-operative; w: week; m: month; “__” indicates medium; “--->” indicates 25th quartile; “–>” indicates 75th quartile.

Similar CGD VAS at baseline was shown between group S (*n* = 42) and group C (*n* = 22) (6.7 ± 0.6 in group S, and 6.6 ± 1.0 in group C). In both groups, the CGD VAS expectedly declined starting at post-operative week 1 to post-operative month 12. However, the declining trend was more significant in group S than that in group C during the 12-month follow-up (2.2 ± 1.3 vs. 4.4 ± 1.1, *p* < .0001 and effect size with 1.76 (1.16, 2.35) post-operative 1st week; 2.1 ± 1.3 vs. 4.9 ± 1.2, *p* < .0001 and effect size with 2.15 (1.52, 2.78)post-operative 1st month; 2.9 ± 1.7 vs. 4.4 ± 1.6, *p* = .0004 and effect size with 0.92 (0.38,1.46)post-operative 3rd month; 3.2 ± 1.6 vs. 4.5 ± 1.5, *p* = .0031 and effect size with 0.78 (0.25, 1.31) post-operative 6th month; 3.1 ± 1.7 vs. 4.5 ± 1.8, *p* = .0011 and effect size with 0.81 (0.27, 1.36) post-operative 12th month, respectively) (Table 3 and Figure 3(a)).

**Table 3.** Comparison of CGD VAS, neck pain VAS and CGD score between two groups in 12-month follow-up.

	Baseline	Post-1w	1 m	3 m	6 m	12 m
CGD VAS						
Group-S (*n* = 42)	6.7 ± 0.6	2.2 ± 1.3	2.1 ± 1.3	2.9 ± 1.7	3.2 ± 1.6	3.1 ± 1.7
Group-C (*n* = 22)	6.6 ± 1.0	4.4 ± 1.1	4.9 ± 1.2	4.4 ± 1.6	4.5 ± 1.5	4.5 ± 1.8
*p* Value	.6939	<.0001	<.0001	.0004	.0031	.0011
Effect size	−0.08 (–0.60, 0.44)	1.76 (1.16, 2.35)	2.15 (1.52, 2.78)	0.92 (0.38, 1.46)	0.78 (0.25, 1.31)	0.81 (0.27, 1.36)
Pain VAS						
Group–S (*n* = 42)	5.8 ± 0.9	2.2 ± 1.3	2.2 ± 1.4	2.9 ± 1.5	3.1 ± 1.4	3.2 ± 1.2
Group-C (*n* = 22)	5.7 ± 1.0	3.8 ± 0.8	4.1 ± 0.8	4.2 ± 1.2	4.2 ± 1.2	4.2 ± 1.3
*p* Value	.9998	<.0001	.0001	.0004	.0043	.0124
Effect size	–0.11 (–0.62, 0.41)	1.32 (0.76, 1.88)	1.54 (0.96, 2.12)	0.93 (0.39, 1.47)	0.86 (0.33, 1.40)	0.76 (0.21, 1.31)
CGD Score						
Group-S (*n* = 42)	4.0 ± 0.6	–	2.2 ± 1.0	2.3 ± 1.0	2.7 ± 1.1	2.6 ± 1.0
Group-C (*n* = 22)	4.0 ± 0.9	–	3.4 ± 0.9	3.3 ± 1.1	3.0 ± 1.3	3.2 ± 1.3
*p* Value	>.9999	–	<.0001	.0009	.7721	.1123
Effect size	–0.03 (–0.56, 0.49)	–	1.17 (0.60, 1.73)	0.90 (0.35, 1.45)	0.45 (–0.08, 0.98)	0.34 (–0.19, 0.87)

Effect size: cohen’s d (95% CI).

CGD: cervicogenic dizziness; VAS: visual analog scale; w: week; m: month.

### Neck pain VAS

No significant difference was found in neck pain VAS between group S (*n* = 42) and group C (*n* = 22) (5.8 ± 0.9 in group S, and 5.7 ± 1.0 in group C). After treatment, both groups showed a recovery trend from post-operative 1st week to post-operative 12th month. Furthermore, remarkable improvement was observed in group S compared to group C (2.2 ± 1.3 vs. 3.8 ± 0.8, *p* < .0001 and effect size with 1.32 (0.76, 1.88) post-operative 1st week; 2.2 ± 1.4 vs. 4.1 ± 0.8, *p* < .0001 and effect size with 1.54 (0.96, 2.12) post-operative 1st month; 2.9 ± 1.5 vs. 4.2 ± 1.2, *p* = .0004 and effect size with 0.93 (0.39, 1.47) post-operative 3rd month; 3.1 ± 1.4 vs. 4.2 ± 1.2, *p* = .0043 and effect size with 0.86 (0.33, 1.40) post-operative 6th month; 3.2 ± 1.2 vs. 4.2 ± 1.3, *p* = .0124 and effect size with 0.76 (0.21, 1.31) post-operative 12th month, respectively) (Table 3 and Figure 3(b)).

### CGD frequency score

A similar baseline score was shown in group S (*n* = 42) and group C (*n* = 22) (4.0 ± 0.6 in group S, and 4.0 ± 0.9 in group C). From post-operative 1st week to post-operative 12th month, a declining trend was found in both groups. Compared with group C, a higher score was reported in group S at post-operative 1st month and 3rd month 2.2 ± 1.0 vs. 3.4 ± 0.9, *p* < .0001 and effect size with 1.17 (0.60, 1.73); and 2.3 ± 1.0 vs. 3.3 ± 1.1, *p* = .0009 and effect size with 0.90 (0.35, 1.45), respectively), but no difference was shown at post-operative 6st month and 12rd month (2.7 ± 1.1 vs. 3.0 ± 1.3, *p* = .7721 and effect size with 0.45 (-0.08, 0.98); and 2.6 ± 1.0 vs. 3.2 ± 1.3, *p* = .1123 and effect size with 0.34 (-0.19, 0.87), respectively) (Table 3 and Figure 3(c)).

#### CGD alleviation rating

To describe the CGD alleviation rating at 12 months post-operative, we applied the Mann–Whitney test as an effective analytic method. Significantly better outcomes were observed in group S compared with group C, *p* = .0005 and effect size with −1.008 (-1.55, −0.44) (Figure 3(d)).

## Complications

Five patients experienced ecchymoma at the needle insertion site and one patient complained of hoarseness. All patients fully recovered by post-operative week 2. No other complications were reported from either group.

## Discussion

Although cervical disc degeneration has been identified as an important pathological origin of CGD – by a series of studies from clinical observation to immunohistochemical staining [13,14], there is little evidence supporting that coblation discoplasty can alleviate CGD in degenerative cervical disc disease. In this study, compared with aconservative intervention approach, coblationdiscoplastyshowed promising therapeutic effects on CGD through a tremendous improvement with an effect size of 1.76, 2.15, 0.92, 0.78 and 0.81 in CGD VAS at post-operative 1 week, and 1, 3, 6, 12 months, respectively.

In this study, we focussed on the “lightheadedness or disequilibrium” sensations as the main symptom of CGD, because the “rotatory” sensation is more closely linked to vestibular disease [20,21]. To target the muscle spindles in the sub-occipital muscles and the Ruffini corpuscles of the cervical joint in the cervico-vestibular pathway, we respectively performed trigger point injections [22] and a cervical medial branch block [23]. This was done to rule out the corresponding sensorimotor control dysfunction. Because inflammation or mechanical compression may disrupt the connection between the cervical nerve root and the vestibular nucleus [5,12], cervical nerve root block was adopted to alleviate dizziness before coblation discoplasty in our study. Subsequently, the pathological source of CGD was identified by intradiscal injection with ≥50% dizziness relief through anaesthetising the nociceptive inputs from mechanoreceptor endings inside [9,17].

As one of the therapeutic mechanisms, the ablation of the intradiscal Ruffini corpuscles aims to disrupt the abnormal proprioceptive inputs from the degenerative cervical disc into the cervico-vestibular pathway. However, unlike the ablation region, mainly located on the centre of the nucleus in Li’s study [18], we focussed on 3 regions during the ablation procedure: the annulus margin, the boundary between the annulus and nucleus, and the midpoint of the nucleus. This is because the Ruffini corpuscles and nociceptors are widely distributed from the outer annulus into the nucleus [13,14,25]. In order to avoid ablating asymptomatic degenerative discs, we chose the single disc as a therapeutic target and a diagnostic method of intradiscal injection, which is different from Li’s study that implemented ablation of double or triple discs [18].

Postural deviations and imbalance are related to pain-related muscular contractions and facet joint abnormal positions [26,27]. As a nociceptive stimulus, pain can alter the function of mechanoreceptors of the muscle spindle (especially in the upper cervical region), and further interfere with the neurophysiological connections between the visual and vestibular systems [28,29]. If an abnormal head and neck orientation was present in the central nervous system (CNS), the cervical muscle would lose control of afferent cervical activity in multiple degrees [30]. Therefore, pain alleviation should be one potential contributor to the CGD recovery after ablation of intradiscal nociceptors and decompression of the nerve root. And another potential therapeutic mechanism should be attributed to the elimination of abnormal mechanoreceptive signal transport in the cervico-vestibular pathway due to decompression of nerve root [5,12].

In addition, because of the unsatisfactory therapeutic efficacy in CGD, 5 patients in group C opted to receive coblation discoplasty. Three of them experienced satisfactory improvement throughout the next 12 months, further identifying that there is a link between dizziness and cervical degenerative disc [31] and supporting that coblation discoplasty is an effective procedure to treat CGD [18]. In addition, due to undesirable coblation outcomes, 3 patients in group S received open surgery and experienced significant dizziness relief. Dizziness should benefit from the total removal of intradiscal mechanoreceptors and nociceptors and complete decompression of the nerve root [13,14,16,17].

Up to the present day, there is no consensus about the link between cervical disc segment and dizziness. In this study, the highest percentage of “dizziness” is C5/6(40%), followed by C4/5(31%) and C3/4(16%), which is consistent with the percentage distribution of “dizziness” disc described in previous clinical studies [13,14,16]. However, the upper cervical discs are considered the major source of dizziness [17], because the C2 and C3 spinal ganglions possess the highest density of afferent fibres projected into the vestibular nucleus [5]. To explain CGD relief after treating lower cervical discs, three reasons are as followed: (1) lower cervical discs are innervated by the neurons in C2 and C3 spinal ganglions [15], (2) lower spinal ganglions possess afferent fibres projected into the vestibular nucleus, even though they are lower density [5], (3) degenerative lower cervical discs possess a positive association with increased number of Ruffini corpuscles [13].

There are some limitations that should be acknowledged. First, we did not perform histologic examination and immunohistochemical analysis for therapeutic disc because removing and collecting the specimens of degenerative cervical discs during coblation procedure is hard to achieve. Second, compared with our retrospective study, randomized clinical controlled trials can more accurately evaluate the outcomes of coblation discoplasty for CGD. Further basic and clinical studies are needed to unravel the correlation between CGD and degenerative cervical disc.

In conclusion, our findings show that there is a significant recovery trend inthe severity and frequency of CGD after coblation discoplasty, which is an effective minimally invasive procedure to bridge unresponsive conservative intervention and open surgery.

## Abbreviations

CGD: cervicogenic dizziness
VAS: visual analogue scale
ITT: intent-to-treat

## References

[1] Takahashi S. Importance of cervicogenic general dizziness. J Rural Med. 2018;13(1):48–56. 29875897 10.2185/jrm.2958 PMC5981019

[2] Jung FC, Mathew S, Littmann AE, et al. Clinical decision making in the management of patients with cervicogenic dizziness: a case series. J Orthop Sports Phys Ther. 2017;47(11):874–884. 28992773 10.2519/jospt.2017.7425

[3] Bisdorff A, Von Brevern M, Lempert T, et al. Classification of vestibular symptoms: towards an international classification of vestibular disorders. J Vestib Res. 2009;19(1–2):1–13. 19893191 10.3233/VES-2009-0343

[4] Devaraja K. Approach to cervicogenic dizziness: a comprehensive review of its aetiopathology and management. Eur Arch Otorhinolaryngol. 2018;275(10):2421–2433. 30094486 10.1007/s00405-018-5088-z

[5] Bankoul S, Goto T, Yates B, et al. Cervical primary afferent input to vestibulospinal neurons projecting to the cervical dorsal horn: an anterograde and retrograde tracing study in the cat. J Comp Neurol. 1995;353(4):529–538. 7539013 10.1002/cne.903530405

[6] Hawk C, Khorsan R, Lisi AJ, et al. Chiropractic care for nonmusculoskeletal conditions: a systematic review with implications for whole systems research. J Altern Complement Med. 2007;13(5):491–512. 17604553 10.1089/acm.2007.7088

[7] Karlberg M, Persson L, Magnusson M. Impaired postural control in patients with cervico-brachial pain. Acta Otolaryngol Suppl. 1995;520 Pt 2:440–442. 10.3109/00016489509125293 8749184

[8] Colledge NR, Barr-Hamilton RM, Lewis SJ, et al. Evaluation of investigations to diagnose the cause of dizziness in elderly people: a community based controlled study. BMJ. 1996;313(7060):788–792. 8842072 10.1136/bmj.313.7060.788 PMC2352174

[9] Sterling M, Jull G, Vicenzino B, et al. Development of motor system dysfunction following whiplash injury. Pain. 2003;103(1):65–73. 12749960 10.1016/s0304-3959(02)00420-7

[10] Peng B. Cervical vertigo: historical reviews and advances. World Neurosurg. 2018;109:347–350. 29061460 10.1016/j.wneu.2017.10.063

[11] McLain RF. Mechanoreceptor endings in human cervical facet joints. Spine. 1994;19:495–501. 8184340 10.1097/00007632-199403000-00001

[12] McCall AA, Miller DM, Yates BJ. Descending influences on vestibulospinal and vestibulosympathetic reflexes. Front Neurol. 2017;8:112. 28396651 10.3389/fneur.2017.00112 PMC5366978

[13] Yang L, Yang C, Pang X, et al. Mechanoreceptors in diseased cervical intervertebral disc and vertigo. Spine. 2017;42(8):540–546 27438387 10.1097/BRS.0000000000001801

[14] Yang L, Chen J, Yang C, et al. Cervical intervertebral disc degeneration contributes to dizziness: a clinical and immunohistochemical study. World Neurosurg. 2018;119:e686–e693. 30092465 10.1016/j.wneu.2018.07.243

[15] Fujimoto K, Miyagi M, Ishikawa T, et al. Sensory and autonomic innervation of the cervical intervertebral disc in rats: the pathomechanics of chronic discogenic neck pain. Spine. 2012;37(16):1357–1362. 22310098 10.1097/BRS.0b013e31824ba710

[16] Peng B, Yang L, Yang C, et al. The effectiveness of anterior cervical decompression and fusion for the relief of dizziness in patients with cervical spondylosis: a multicentre prospective cohort study. Bone Joint J. 2018;100-B(1):81–87. 29305455 10.1302/0301-620X.100B1.BJJ-2017-0650.R2

[17] Yi YY, Xu HW, Zhang SB, et al. Does the C3/4 disc play a role in cervical spondylosis with dizziness? A retrospective study. Int Ortho. 2020;44(6):1159–1168. 10.1007/s00264-020-04531-y 32193610

[18] Li S, Chen R, Chen Y, et al. Therapeutic effects and safety of percutaneous disc decompression with coblation nucleoplasty in cervical vertigo: a retrospective outcome study with 74 consecutive patients and minimum 1-year follow-up. Pain Phys. 2019;22:E205–E214. 31151343

[19] He L, Tang Y, Li X, et al. Efficacy of coblation technology in treating cervical discogenic upper back pain. Medicine. 2015;94(20):e858. 25997062 10.1097/MD.0000000000000858 PMC4602856

[20] Reid SA, Callister R, Katekar MG, et al. Utility of a brief assessment tool developed from the Dizziness Handicap Inventory to screen for cervicogenic dizziness: a case control study. Musculoskelet Sci Pract. 2017;30:42–48. 28521181 10.1016/j.msksp.2017.03.008

[21] Spiegel R, Rust H, Baumann T, et al. Treatment of dizziness: an interdisciplinary update. Swiss Med Wkly. 2017;147:w14566. 29282702 10.4414/smw.2017.14566

[22] Baron EP, Cherian N, Tepper SJ. Role of greater occipital nerve blocks and trigger point injections for patients with dizziness and headache. Neurologist. 2011;17(6):312–317. 22045281 10.1097/NRL.0b013e318234e966

[23] Hahn T, Halatsch ME, Wirtz C, et al. Response to cervical medial branch blocks in patients with cervicogenic vertigo. Pain Phys. 2018;21(3):285–294. 29871373

[24] Shah PB. Intention-to-treat and per-protocol analysis. CMAJ. 2011;183(6):696; author reply 696. 10.1503/cmaj.111-2033 PMC3071397 21464181

[25] Wu B, Yang L, Peng B. Ingrowth of nociceptive receptors into diseased cervical intervertebral disc is associated with discogenic neck pain. Pain Med. 2019;20(6):1072–1077. 30848823 10.1093/pm/pnz013

[26] Yaseen K, Hendrick P, Ismail A, et al. The effectiveness of manual therapy in treating cervicogenic dizziness: a systematic review. J Phys Ther Sci. 2018;30(1):96–102. 29410575 10.1589/jpts.30.96 PMC5788784

[27] Bittar R, Alves NG, Bertoldo C, et al. Efficacy of carbon microcoils in relieving cervicogenic dizziness. Int Arch Otorhinolaryngol. 2017;21(1):4–7. 28050200 10.1055/s-0036-1592418 PMC5205517

[28] L'Heureux-Lebeau B, Godbout A, Berbiche D, et al. Evaluation of paraclinical tests in the diagnosis of cervicogenic dizziness. Otol Neurotol. 2014;35(10):1858–1865. 25058834 10.1097/MAO.0000000000000506

[29] Kristjansson E, Treleaven J. Sensorimotor function and dizziness in neck pain: implications for assessment and management. J Orthop Sports Phys Ther. 2009;39(5):364–377. 19411769 10.2519/jospt.2009.2834

[30] Li Y, Peng B. Pathogenesis, diagnosis, and treatment of cervical vertigo. Pain Physician. 2015;18(4):E583–95. 26218949

[31] Micarelli A, Viziano A, Augimeri I, et al. Diagnostic route of cervicogenic dizziness: usefullness of posturography, objective and subjective testing implementation and their correlation. DisabilRehabil. 2019;1–8. 10.1080/09638288.2019.1680747 31656108

